# Maternal Immunoglobulins in Infants—Are They More Than Just a Form of Passive Immunity?

**DOI:** 10.3389/fimmu.2020.00855

**Published:** 2020-05-19

**Authors:** Kateryna Pierzynowska, Jarosław Woliński, Björn Weström, Stefan G. Pierzynowski

**Affiliations:** ^1^Department of Animal Physiology, The Kielanowski Institute of Animal Physiology and Nutrition, Polish Academy of Sciences, Jabłonna, Poland; ^2^Department of Biology, Lund University, Lund, Sweden; ^3^SGP + GROUP, Trelleborg, Sweden; ^4^Department of Medical Biology, Institute of Rural Health, Lublin, Poland

**Keywords:** immunoglobulins, extra-immunological effects, neonatal, brain development, gut development

## Abstract

In the present review, we highlight the possible “extra-immunological” effects of maternal immunoglobulins (Ig) transferred to the blood circulation of offspring, either via the placenta before birth or via the colostrum/milk across the gut after birth in different mammalian species. Using the newborn pig as a model, since they are naturally born agammaglobulinemic, intravenously (i.v.) infused purified serum Ig rapidly improved the vitality, suckling behavior, and ensured the survival of both preterm and term piglets. In further studies, we found that proper brain development requires i.v. Ig supplementation. Studies have reported on the positive effects of i.v. Ig treatment in children with epilepsy. Moreover, feeding newborn pigs an elementary diet supplemented with Ig improved the gut structure, and recently a positive impact of enteral or parenteral Ig supplementation on the absorption of polyunsaturated fatty acids (PUFAs) was observed in the newborn pig. Summarized, our own results and those found in the literature, indicate the existence of important extra-immune effects of maternal Ig, in addition to the classical protective effects of transferred maternal passive immunity, including effects on the development of the brain, gut, and possibly other organ systems in the neonate. These additional properties of circulating Ig could have an impact on care guidelines for human neonates, especially those born prematurely with low plasma Ig levels.

## Introduction

Human infants receive the majority of maternal immunoglobulins (Ig), predominantly immunoglobulin G (IgG), via the placenta. Maternal antibody transfer to the fetus starts as early as during the 13th week of gestation. The level of IgG in the fetal circulation is relatively low (5–10% of maternal levels) between weeks 17 and 22, reaching 50% of maternal levels by week 32 and usually exceeding the maternal plasma IgG level at birth (1).

In ungulate species, in contrast to humans, Ig are exclusively transferred from the mother to the newborn after birth through the ingestion of the “first milk” or colostrum, which is rich in IgG. The Ig are transferred across the “open” gut to the general circulation during the first few days of life. Thus, since piglets are born agammaglobulinemic, they serve as an excellent model for studying the biological effects of maternal Ig transfer in the newborn. In addition, since the pig is multiparous and may give birth to 10–20 piglets at a time, it is possible to form several treatment groups from one litter, making comparative studies easier.

Newborn, unsuckled piglets are in a biological sense transiently immunodeficient, and Ig are not always optimally transmitted from mother to offspring, e.g., because of premature delivery in humans or problems with suckling in ungulates. This raises the question as to whether a transient immunodeficiency could result in symptoms similar to those observed in classical primary immunodeficiency (PID) later in life. Patients with classical PID, as a result of various gene defects, develop various immune disorders later in life (2) with variable clinical manifestations, e.g., autoimmune disorders and malignancies or allergic disorders (3).

## Maternal Ig Influence on the Structural and Functional Development of the Brain

Maternal Ig, transferred to the offspring's blood stream, either before birth (in the case of human infants) or after birth (in the case of ungulates), are omnipresent and may function as regulators for those organs and systems undergoing drastic developmental changes after birth, e.g., the gut, brain, immune system, etc. However, any extra-immune regulatory effects of Ig are difficult to recognize at “first glance,” and are probably often overlooked because of “the most obvious role of Ig in protecting the offspring from infections during the first few weeks of life.” Hence, the aim of our review is to highlight and summarize the knowledge obtained from previous studies by our lab, as well as previous literature concerning the possible extra-immunological effects of maternal Ig transfer during the early post-natal period and their (Ig) role in further development of the offspring.

In the case of livestock praxis with regard to goat breeding, it is well-known that careful surveillance of the pasture should be carried out during delivery time in order to locate newborn kids. If the kids do not receive colostrum containing high levels of IgG timeously, they become apathetic and die within a few hours after birth, with no obvious symptoms of infection (4). Similarly, pig breeders usually keep some colostrum in reserve in order to vitalize piglets, which are not able to suckle from their mothers. Nowadays, sows, which have a maximum of 14 teats, usually give birth to over 20 piglets. Thus, in order for all the piglets to survive (5), breeders need to feed the surplus piglets Ig-rich colostrum collected from other sows or from cows. If piglets do not get a sufficient amount of colostrum for absorption over the gut during the first few hours of life, they become apathetic, often have watery diarrhea, and finally die—generally not as a result of infection.

To study this inexplicable effect of colostrum, we performed some simple experiments to determine whether provision of purified Ig, the major protein in colostrum whey, using different approaches, would be beneficial for term and preterm newborn piglets (6). A preparation of porcine serum Ig was infused i.v. in an amount sufficient to ensure the attainment of blood levels of IgG similar to those found in piglets fed with sow colostrum. Infusion of the Ig preparation in both preterm and term newborn (un-suckled) piglets ensured their active suckling behavior, growth, and survival, as well as blood IgG and protein levels similar to those observed in piglets fed colostrum. In contrast, piglets completely deprived of Ig exhibited no willingness to suckle and exhibited very low blood levels of IgG and lower protein levels compared to colostrum-fed or Ig-infused animals. Moreover, piglets infused with sow serum, containing less IgG than the Ig preparation, displayed significantly lower blood IgG levels, compared to those infused with the Ig preparation or those fed colostrum, and did not develop proper suckling behavior. In conclusion, the experiments suggest that early systemic infusion of Ig is key to stimulating behavioral survival instincts and ensuring the well-being of newborn piglets, either preterm or full term. In addition, the experiments indicated that the agammaglobulinemic newborn pig can be used as an animal model for the human infant.

Neurological disorders are among the main clinical problems affecting preterm children and often cause communication problems and learning disabilities later on in life (7, 8). Several factors are important for brain development, but the role of maternal Ig transfer has not yet been investigated with regard to this aspect. The first results indicating positive effects of Ig administration on protein synthesis in the brain were obtained by Burrin et al. (9). Colostrum was found to be the best stimulator of protein synthesis in vital organs of newborn piglets, e.g., the brain and heart, compared to an isoenergetic diet of milk formula or mature milk. The authors attributed the stimulation of specific protein synthesis in the brain and heart to the colostrum that was fed to the piglets, and not to the intake of certain macronutrients. These findings suggested to us that it was the high content of Ig in the colostrum that had the stimulatory effect on brain growth, development and metabolism. Moreover, Harada et al. (10) have shown that following colostrum feeding, the IgG can penetrate into the cerebrospinal fluid (CSF) of neonatal piglets.

Thus, the main objective in a study performed in our lab (11), was to evaluate the effects of colostrum (Ig) on brain development in neonatal pigs during the first 3 days after birth. Positive correlations were found between growth and hippocampal development and the levels of total protein and IgG in blood plasma of sow-reared piglets. Piglets that were exclusively fed an elemental diet exhibited reduced counts of microglial cells and neurons in the CA1 area of the hippocampus 72 h after birth. However, supplementation of an elemental diet with Ig or rearing the piglets with sow colostrum improved the cellular structure and supported the trophic status of the hippocampus. The data obtained indicated that the development of the hippocampus requires Ig in order to stimulate protein synthesis and brain development during the early post-natal period. In order to focus on the specific role of IgG, further investigations are required, since the Ig preparation used in our studies contained a mixture of Ig classes. However, though colostrum contains several Ig classes, predominantly IgG is absorbed over the gut before gut closure (12).

In a follow-up study by our lab, newborn piglets were fed an infant formula or colostrum supplemented, orally or i.v., with either species-specific, porcine, or foreign, human, Ig and compared to newborn un-suckled piglets or sow-reared piglets (13). After 2 days, behavioral tests were performed on the piglets. Both neuronal plasticity parameters, i.e., neuronal maturation and synapse-associated proteins, and behavioral test parameters seemed to only be improved by the presence of the species-specific porcine Ig in the circulation and in the cerebrospinal fluid (CSF). In fact, Kowal et al. (14) have discussed the possible role of Ig on the development of the blood–brain barrier in humans. Why during the early stages of development are Ig permitted to enter the CSF, since the presence of Ig in the CSF of adults is considered a pathological sign? An indication might be that the presence of Fc gamma receptors have been described in the developing rat brain (15, 16) and the same type of Fc receptor is known to be of importance in the development of the cerebellum (17). Despite the expression profile and functionality of Fc receptors in neurons being not well-investigated and controversial, there is accumulating evidence that all four types of Fc receptors are expressed in neurons (17–22). Moreover, stimulation of mouse superior cervical ganglions with IgG *in vitro* leads to an increase in intracellular calcium (18). Thus, maternal Ig appear to play some sort of regulatory role, e.g., enabling the transfer of maternal experience to the developing brain.

## Maternal Ig and the Development of Epilepsy

A positive influence of i.v. Ig treatment on epilepsy in children, with a decrease in frequency and severity of seizures, was observed as early as 1977 by Pechadre et al. (23). At the time, this finding supported the allergic theory of epilepsy and was recognized as a form of immunological treatment. However, both animal and human studies suggest non-immunological effects of i.v. Ig treatment. Patients with both idiopathic and symptomatic forms of epilepsy demonstrated an immediate response to Ig infusion. I.v. Ig infusion has also been shown to have anticonvulsant effects in the kindled cat model (24) and in a model of direct cortical stimulation, where i.v. treatment with Ig significantly decreased seizure threshold (25). All these observations suggest a direct neuro-modulatory effect of i.v. Ig treatment (25).

It has been reported that an increase in gestational age is negatively correlated with the risk of epilepsy (26). Taking into consideration that i.v. Ig treatment has been used in different forms of the intractable childhood epilepsy with promising results (up to 70% of patients obtaining a seizure-free status) (27, 28), and the fact that plasma levels of IgG are positively correlated with gestational age increase (1), one should consider Ig as possible neuromodulators, which regulate excitability of neuronal membranes and protect the immature brain of newborn infants against over excitation. Furthermore, Ig have been shown to be taken up by neurons (20), causing direct neuroprotective effects via the modulation of NF-kB and MAPK activities, through the reduced expression and activation of neuronal toll-like receptors, as well as by decreasing caspase-3 cleavage leading to decreased apoptosis of neurons (29–32).

We postulate possible positive clinical effects of i.v. infusion of human Ig in terms of stimulating neuronal plasticity and development of cognitive function in preterm infants born with low immunoglobulin levels in their blood. This is supported by the review by Chavoshzadeh et al. (33) who postulate that early recognition and treatment of primary immunodeficiencies (PID) is important to prevent or reduce future irreversible neurological sequelae. Diverse neurological deficits accompanying certain PID may be mild. However, they may greatly influence the course of the disease with major impacts on the quality of life of these patients.

## Maternal Ig Support Structural and Functional Development of the Gut

Nowadays, a lot of research is performed on the influence of the gut microbiota on infant development and metabolism, a lot of which involves maternal Ig derived from mother's milk or retro-transported via the FcRn receptor from the serum to the gut (34). Gut microbiota develops via interactions with nutrients (35), both endogenous and exogenous agents, such as melatonin (36) or fructans (37), and even viral infections (38), with involvement of maternal and infant Ig. Recently (39), it has been shown that maternal IgA decreases the risk of development of necrotizing enterocolitis through its (IgA) influence on the host–microbiota relationship in preterm neonates. However, all these investigations focus on the classical functions of the immune system and its impact on overall gut function.

To better address this issue, we investigated whether Ig administration affects the structure of the gastrointestinal tract in newborn piglets (40). Enteral feeding with an elementary diet supplemented with purified Ig resulted in a significant increase in the thickness of the stomach, duodenal and jejunal mucosa, and muscularis layers compared to that observed in the group fed an elementary diet without Ig. The parameters measured in the Ig-fed group reached values similar to those observed in sow-reared piglets. Finally, colostrum and Ig may have a protective effect via blockage of the pro-inflammatory reaction of the enteral nervous system (40). Our results show that a diet supplemented with Ig stimulates growth of the gut and affects intestinal structure by altering it toward that observed in colostrum-fed piglets, which indicates a direct beneficial effect of Ig on gut development in neonatal pigs.

In a recent study, we investigated the impact of Ig on intestinal function and the absorption of polyunsaturated fatty acids (PUFAs) in the newborn pig (41). The high plasma levels of PUFAs found in newborn, un-suckled piglets decrease by between 40 and 50% in piglets fed an infant formula for 48 h. However, piglets fed the infant formula supplemented with Ig, either orally or through feeding with swine or bovine colostrum, or by swine serum infusion, or by i.v. infusion of swine or human Ig preparations, demonstrated improved growth and enhanced plasma PUFA levels, similar to those observed at birth. These results indicate the importance of the presence of Ig in the blood for the appropriate absorption of dietary PUFAs and overall gut function and, thus, the absorption of other nutrients in newborn piglets. This may have an impact on the dietary guidelines for human neonates, especially those born prematurely with low plasma Ig levels, since PUFAs are important factors for brain development in early life.

## Implications

The extra-immunological effects of circulating maternal Ig, presumably more specifically IgG, in the newborn are summarized in this review highlighting the importance of maternal Ig transfer in stimulating organ growth and maturation after birth. The classical immune-protecting features of maternal Ig can be replaced by, e.g., antibiotics and sterile conditions, while the extra-immunological stimulatory effects probably cannot be replaced. Thus, the properties and role of maternal Ig transmitted to the offspring in late pregnancy or directly after birth need to be further explored and recognized as vital components of early development with possible long-lasting effects on health and performance later in life.

## Author Contributions

KP, JW, BW, and SP were responsible for preparing the manuscript. KP, SP, and BW designed the review and critically reviewed the manuscript. All authors contributed to manuscript revision, have read, and approved the submitted version.

## Conflict of Interest

SP is the owner of SGP+ Group, Sweden; KP is employed by the SGP+ Group, Sweden. The remaining authors declare that the research was conducted in the absence of any commercial or financial relationships that could be construed as a potential conflict of interest.
